# *Aeromonas hydrophila* OmpW PLGA Nanoparticle Oral Vaccine Shows a Dose-Dependent Protective Immunity in Rohu (*Labeo rohita*)

**DOI:** 10.3390/vaccines4020021

**Published:** 2016-06-01

**Authors:** Saurabh Dubey, Kiran Avadhani, Srinivas Mutalik, Sangeetha Madambithara Sivadasan, Biswajit Maiti, Joydeb Paul, Shivani Kallappa Girisha, Moleyur Nagarajappa Venugopal, Stephen Mutoloki, Øystein Evensen, Indrani Karunasagar, Hetron Mweemba Munang’andu

**Affiliations:** 1 Department of Fisheries Microbiology, Karnataka Veterinary, Animal & Fisheries Sciences University, College of Fisheries, Mangalore 575002, India; saurabh.dubey@nmbu.no (S.D.); sangeethasivadas888@gmail.com (S.M.S.); skgirisha@gmail.com (S.K.G.); mnvenu@rediffmail.com (M.N.V.); 2 Section of Aquatic Medicine and Nutrition, Department of Basic Sciences and Aquatic Medicine, Faculty of Veterinary Medicine, Norwegian University of Life Sciences, Adamstuen Campus, Ullevålseveien 72, P.O. Box 8146, NO-0033 Dep, Oslo 0454, Norway; joydeb.paul@nmbu.no (J.P.); stephen.mutoloki@nmbu.no (S.M.); oystein.evensen@nmbu.no (Ø.E.); 3 Department of Pharmaceutics, Manipal College of Pharmaceutical Sciences, Manipal University, Manipal 576104, Karnataka State, India; kirankle84@gmail.com (K.A.); ssmutalik@yahoo.com (S.M.); 4 UNESCO MIRCEN for Medical and MarineBiotechnology, Nitte University Centre for Science Education and Research, Nitte University, Deralakatte, Mangalore 575018, India; maitibiswajit@yahoo.com (B.M.); karuna8sagar@yahoo.com (I.K.)

**Keywords:** *Aeromonas hydrophila*, rohu, PLGA, nanoparticle, oral, outer membrane protein

## Abstract

*Aeromonas hydrophila* is a Gram-negative bacterium that causes high mortality in different fish species and at different growth stages. Although vaccination has significantly contributed to the decline of disease outbreaks in aquaculture, the use of oral vaccines has lagged behind the injectable vaccines due to lack of proven efficacy, that being from primary immunization or by use of boost protocols. In this study, the outer membrane protein W (OmpW) of *A. hydrophila* was cloned, purified, and encapsulated in poly d,l-lactide-*co*-glycolic acid (PLGA) nanoparticles (NPs) for oral vaccination of rohu (*Labeo rohita* Hamilton). The physical properties of PLGA NPs encapsulating the recombinant OmpW (rOmpW) was characterized as having a diameter of 370–375 nm, encapsulation efficiency of 53% and −19.3 mV zeta potential. *In vitro* release of rOmpW was estimated at 34% within 48 h of incubation in phosphate-buffered saline. To evaluate the efficacy of the NP-rOmpW oral vaccine, two antigen doses were orally administered in rohu with a high antigen (HiAg) dose that had twice the amount of antigens compared to the low antigen (LoAg) dose. Antibody levels obtained after vaccination showed an antigen dose dependency in which fish from the HiAg group had higher antibody levels than those from the LoAg group. The antibody levels corresponded with post challenge survival proportions (PCSPs) and relative percent survival (RPS) in which the HiAg group had a higher PCSP and RPS than the LoAg group. Likewise, the ability to inhibit *A. hydrophila* growth on trypticase soy agar (TSA) by sera obtained from the HiAg group was higher than that from the LoAg group. Overall, data presented here shows that OmpW orally administered using PLGA NPs is protective against *A. hydrophila* infection with the level of protective immunity induced by oral vaccination being antigen dose-dependent. Future studies should seek to optimize the antigen dose and duration of oral immunization in rohu in order to induce the highest protection in vaccinated fish.

## 1. Introduction

*Aeromonas hydrophila* is a Gram-negative bacteria that causes hemorrhagic septicemia, dropsy, and mortality in different fish species at different growth stages [1,2]. Vaccination has proved to be an effective disease preventive strategy with ability to reduce disease outbreaks [3,4]. Although injectable vaccines that offer protective immunity have been developed for *A. hydrophila* [5,6], the development of oral vaccines has lagged behind the injectable vaccines due to lack of efficacy and antigen formulations that maintain antigen integrity and immunogenicity [7]. An immunologic adjuvant is any substance that is able to accelerate, prolong, or enhance antigen-specific immune response when used in combination with specific antigens [8]. Adjuvants enhance immunogenicity, reduce the amount of antigen required per dose and also reduce the number of boosters needed for long-term protective immunity [8,9,10,11]. As pointed out by Munang’andu and Evensen [9], adjuvants are designed to serve as antigen delivery vehicles and as immunostimulants that would be able to enhance antigen uptake. The search for oral adjuvants has attracted a lot of interest in biodegradable polymeric nanoparticles (NP) because of their dual ability to serve as antigen delivery vehicles and to permit a sustained release of antigens and a consequent reduction of booster vaccinations [9,12,13,14,15]. Among the polymeric systems, poly d,l-lactide-*co*-glycolic acid (PLGA) NPs have been widely used for the controlled delivery of peptides, synthetic proteins, and nucleic acids in humans [16]. Hence, this immunization strategy is being widely explored for the delivery of oral vaccines in finfish [12,13,14,15].

Given their large size, encapsulation of whole cell bacteria (0.5–5.0 μm diameter) in PLGA NPs (<500 nm), is not practical. Therefore, the practical approach is to identify immunogenic proteins found on bacterial surfaces that are able to induce protective immunity for use as vaccine candidates. These can then be used for encapsulation in PLGA NPs. Bacterial outer membrane proteins (OMPs) are among the potential candidates shown to evoke protective immune responses in vaccinated fish because of their exposed epitopes on cell surfaces [17]. The β-barrel architecture of OMPs is easily recognized by host pattern recognition receptors (PRRs) as pathogen associated molecular patterns (PAMP) [18]. OMPs have been widely studied as vaccine candidates for most of the *Enterobacteriaceae* spp*.* where their structural layout has been shown to play an important role in inducing protective immune responses in vaccinated fish. The objective of the present study was to assess the effect of recombinant *A. hydrophila* OmpW encapsulated in PLGA NP in inducing protection against mortality after oral delivery in rohu (*Labeo rohita*) and whether this effect is dose dependent.

## 2. Materials and Methods

### 2.1. Expression and Purification of A. hydrophila Recombinant OmpW Protein

A recombinant OmpW (rOmpW) clone generated in our lab at Mangalore, India, was expressed in an *Escherichia coli* M15 clone, as previously described [18]. The *A. hydrophila* isolate used for cloning and expression of the OmpW protein was isolated from rohu expressing clinical signs of epizootic ulcerative syndrome (EUS). Morphological, biochemical, and molecular characterization of the isolate have previously been described by Maiti *et al.* [18]. The sequence for the OmpW retrieved from this isolate has been deposited in the National Center for Biotechnology Information (NCBI) databank (accession no. HM063443.1), while its structural properties have also been described previously [18]. For large-scale production, *E. coli* containing the rOmpW clone was inoculated in 200 mL Luria Bertani (LB) broth and induced with 1 mM isopropyl thiogalactoside (IPTG) and purified using affinity chromatography. Expression and purity analysis of the rOmpW protein was done by 15% SDS-PAGE and the concentration measured as described by Lowry *et al.* [19].

### 2.2. Encapsulation of rOmpW in PLGA Nanoparticles

Encapsulation of rOmpW in PLGA nanoparticles (PLGA; 50:50 ratio; inherent viscosity: 0.45–0.6 dL/g; molecular weight (MW): 38,000–54,000 Purac Biomaterials, Montville, NJ, USA) was done using the W_1_/O/W_2_ double emulsion solvent evaporation method described previously [20,21,22,23,24,25,26] with some minor modifications. Briefly, 10 mg of the peptide was dissolved in 2 mL (5 mg/mL) milli-Q water (pH 7.4) and emulsified in 20 mL dichloromethane (DCM) containing 100 mg PLGA using a high-speed homogenizer (Polytron, Kinematica AG, Littau-Luzem, Switzerland) at 16,500 rpm for 5 min on ice. Thereafter, 8 mL of 1% *w*/*v* poly vinyl alcohol solution (PVA; Average MW: 30,000–70,000; 87%–90% hydrolysed; Sigma, St. Louis, MO, USA) was added. Homogenization was carried out for 10 min and the emulsion formed was sonicated at 60 amplitude and 4 s pulse (Vibra Cell, VC 130, Sonics and Materials, Newton, CT, USA) on ice for 30 min. Thereafter, 90 mL 1% *w*/*v* PVA was added to the double emulsion solution formed after sonication. The double emulsion solution was allowed to evaporate at room temperature by keeping the dispersion overnight with stirring using a mechanical stirrer. The particles were separated by centrifugation at 22,000 rpm (4 °C) for 45 min. Finally, the pellet obtained was dispersed in 5% *w*/*v* trehalose solution and subjected to lyophilization. Empty PLGA nanoparticles without rOmpW protein were prepared using the same protocol.

### 2.3. Characterization of PLGA Nanoparticles and In Vitro Release Test

Encapsulation efficiency of rOmpW was determined by dissolving the particles in 0.1 M NaOH containing 0.5% *w*/*v* SDS followed by adjusting the pH to 7.4 [19]. The size and zeta potential of the PLGA nanoparticles after lyophilization was determined using the particle size analyzer (NanoZS, Malvern instruments, Malvern, Worcestershire, UK). An *in vitro* release test of rOmpW was carried out to determine the timing of antigen release from the PLGA nanoparticles by putting 10 mg PLGA nanoparticles encapsulating rOmpW in 500 mL phosphate buffered saline (PBS) in a water bath shaker at 37 °C. *In vitro* release of rOmpW was evaluated by drawing 500 μL supernatant, which was replaced with an equal volume of PBS after centrifugation at 10,000× *g* for 10 min. Samples for *in vitro* release were collected at 1, 2, 8, 16, 24, and 48 h intervals. The released rOmpW protein was quantified using the method of Lowry *et al.* [19].

### 2.4. Vaccine Preparations for Oral Delivery

To prepare the PLGA NPs rOmpW (NP-rOmpW) vaccine for oral immunization, commercial feed for rohu was ground and sieved. The NP-rOmpW vaccine was thoroughly mixed with feed and made into a dough. This was followed by pelletizing the vaccine-feed mixture by pressing through a hand extruder having a diameter of 2 mm. The pellets were dried at room temperature and stored at 4 °C until use.

### 2.5. Vaccination and Challenge

**Figure 1:**
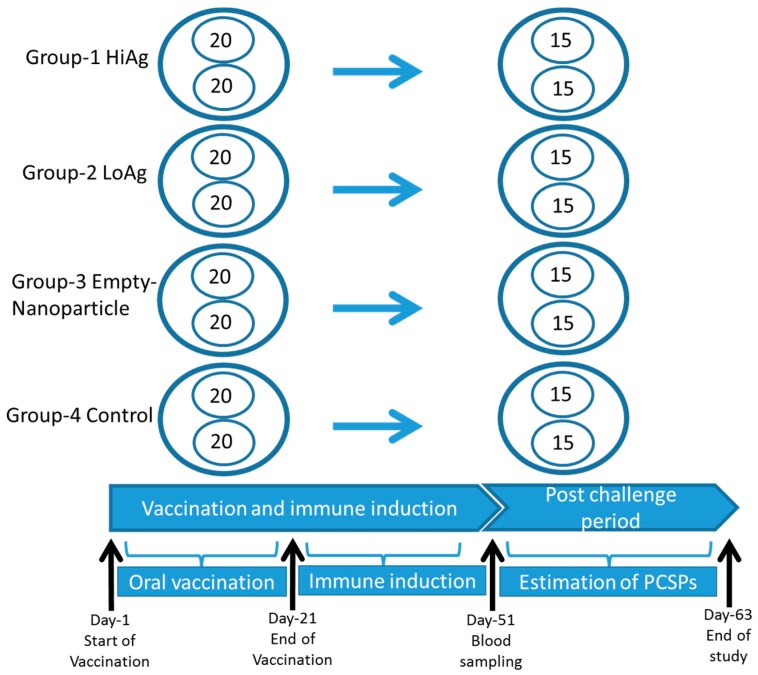
The study design for oral vaccination of rohu using different vaccine against *A. hydrophila*. Four vaccine groups were each allocated 40 fish. Group 1 was allocated a high-antigen dose of 8 μg/g of fish body weight of the rOmpW vaccine designated as the HiAg dose. Group 2 was vaccinated with a low-antigen dose of 4 μg/g of fish body weight of the rOmpW designated as the LoAg dose. Group 3 was vaccinated with empty nanoparticles, without the rOmpW antigen, designated as NP-Empty while Group 4 was left unvaccinated as a control group. The study time-line was segmented into three parts, namely, (i) the oral vaccination period of 21 days; (ii) immune induction period of 50 days post vaccination (dpv); and (iii) post challenge period. Blood samples were collected from 10 fish per group at 50 dpv after which fish were challenged with a virulent strain *A. hydrophila* at a concentration of 2.7 × 10^7^ CFU/mL injected intramuscularly at 0.1 mL/fish. The vaccination trial ended at 83 dpv when fish stopped dying.

Healthy *Labeo rohita* with an average weight of 10 g were brought to the wet lab in oxygenated bags from the college farm, Mangalore, India. Examination of the health status of fish was based on clinical observations by checking for any abnormal appearances and swimming behavior. In addition, six were sacrificed for pathological examination. Both gross pathology and histopathology examination did not show pathological changes. Fish were kept in recirculating water at 28 °C with uniform aeration during acclimatization for a month and fed *ad libitum*. For vaccination, 160 fish were taken and distributed at equal numbers into eight tubs as shown in Figure 1. Group 1 was allocated 40 fish distributed in duplicate tubs, with 20 in each, and was vaccinated with a high-antigen dose of 8 μg/g of fish body weight designated the HiAg group. Likewise, Group 2 was also allocated 40 fish distributed to two tubs and was vaccinated with a low-antigen dose of 4 μg/g of fish body weight designated the LoAg group. Group 3 was fed with empty NPs without the rOmpW antigens, while group 4 was only given feed and left unvaccinated as a control group. The vaccine-coated feed was given to rohu twice daily for 21 days. There was no significant difference observed in feed intake and overall growth in all groups. Blood samples were collected from 10 fish in each group after 30 days post vaccination (dpv). Serum was separated from the blood and stored at −20 °C until use. After 30 dpv, fish were challenged using a pathogenic strain of *A. hydrophila* (Ah40, 2.7 × 10^7^ cfu/mL) [27] by intramuscular injection at 0.1 mL/fish. The *A. hydrophila* isolate used for challenge is similar to the isolate that was used to produce the NP-rOmpW vaccines [18]. Its pathogenicity in fish has been documented in previous challenges studies [27]. Mortalities were recorded and protection was estimated using the Kaplan Meyer’s survival analysis.

### 2.6. Serum Inhibitory Assay

The serum mediated antibacterial activity was measured as described by Hamod *et al.* [17]. Briefly, 10 μL of *A. hydrophila* grown overnight in broth culture was adjusted to 10^3^ cfu/mL in PBS to which 90 μL serum was added in a microtube. The solution was mixed thoroughly and placed at 30 °C for 24 h. After incubation, a 10-fold serial dilution of serum containing bacteria was prepared for each mixture and 100 μL aliquots of each dilution was plated onto tryptone soya agar plates and incubated at 30 °C for 24 h. Bacterial colonies were counted and results were expressed as log_10_ cfu/mL. The serum used for this study was pooled from the 10 fish sampled at 30 dpv from each group described in Section 2.6 above. Reduction in bacterial count was obtained by subtracting the bacterial count of vaccinated fish from counts of PBS control fish.

### 2.7. Antibody Response to the rOmpW Protein

Antibody responses to rOmpW were analyzed using the enzyme linked immunosorbent assay (ELISA). Briefly, ELISA plates (Greiner Bio-One, Frickenhausen, Germany) were coated with 2 μg/well rOmpW diluted in carbonate-bicarbonate buffer (pH 9.6) and incubated overnight at 4 °C. The plates were blocked with 350 μL 3% BSA at 37 °C for 2 h after washing using PBS. Thereafter, 100 μL fish sera (1:10 dilution) was added to each well and incubated at 37 °C for 2 h. The plates were washed three times with PBS, followed by adding rabbit anti-rohu HRPO at 1:200 dilution per well and incubated at 37 °C for 35 min. After washing, tetramethylbenzidine hydrogen peroxide (TMB) substrate was added to each well and the absorbance was read at 450 nm using an EL_X800_ Universal microplate reader (BioTek, Winooski, VT, USA).

## 3. Results

### 3.1. Antigen Preparation

#### 3.1.1. Expression, Purification, Concentration, and Encapsulation Efficiency of OmpW

The rOmpW protein was expressed after 4 h induction with IPTG. The MW was determined to be 22 kDa using 15% SDS-PAGE. The purity of rOmpW was about 90% and the concentration was estimated at 1.2 mg/mL. The encapsulation efficiency of rOmpW was estimated at 53.56% and average particle size diameter was 370–375 nm. The zeta potential of rOmpW encapsulated PLGA nanoparticles was −19.3 mV.

#### 3.1.2. *In Vitro* Release of the rOmpW Protein from PLGA Nanoparticles

**Figure 2:**
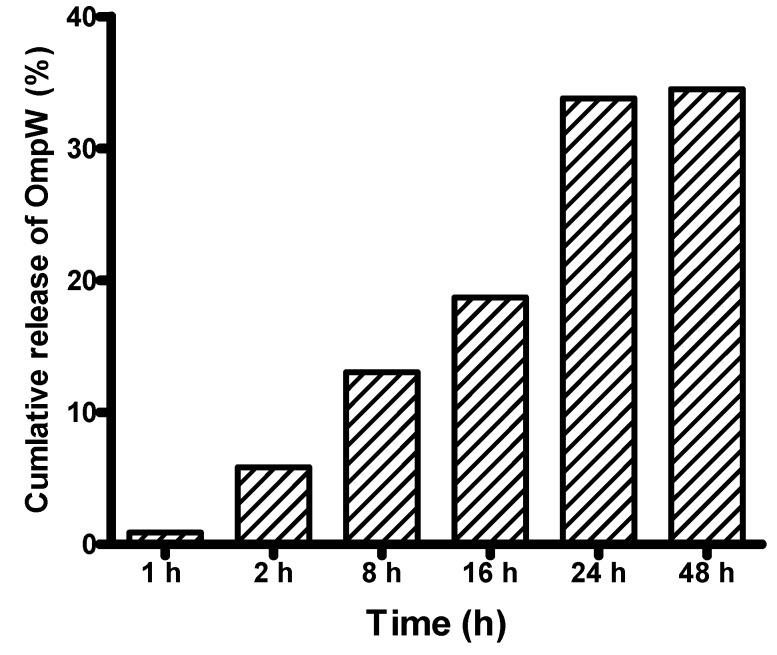
Shows *in vitro* release of rOmpW protein from encapsulated PLGA NPs observed within 48 h of incubation in phosphate buffered saline (PBS). Note that the release of rOmpW increased exponentially within 24 h after the start of the *in vitro* test.

*In vitro* release of rOmpW showed an exponential increase in the first 24 h of incubation (Figure 2), which progressed to the plateau phase after 24–48 h of incubation in PBS.

### 3.2. Vaccination and Challenge of Rohu

#### 3.2.1. Kaplan Meyer’s Survival Analysis

**Figure 3:**
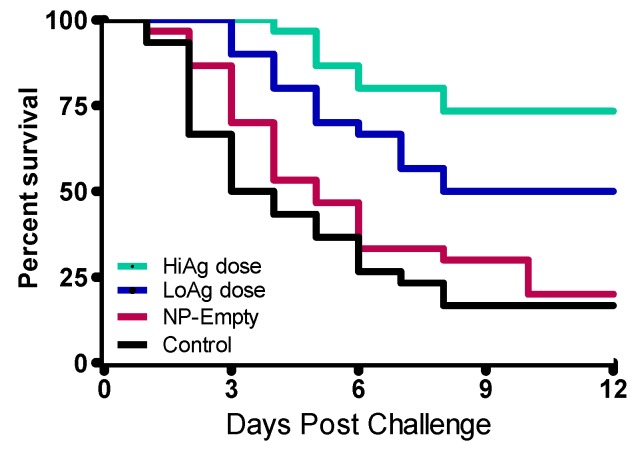
The Kaplan Meyer’s survival analysis of fish vaccinated against *A. hydrophila* using with the NP-rOmpW vaccine administered at high- and low-antigen doses. The high-antigen (HiAg) dose was administered at a concentration of 8 μg/g of fish body weight, while the low-antigen (LoAg) dose was administered at a dose of 4 μg/g of fish body weight. Mortality in the control group started at two days post challenge (dpc), 3 dpc in the LoAg group and 4 dpc in the HiAg group. The highest post challenge survival proportion (PCSPs) were from the HiAg group (PCSP = 73.33%), followed by the LoAg group (PCSP = 48.28%), while the NP-Empty (PCSP = 20.00%) and control (PCSP = 16.67%) groups had the lowest PCSPs. There was a significant difference (*P* = 0.0435) between the HiAg and LoAg groups, while no significant difference (*P* = 0.3104) was observed between the NP-Empty and control groups.

A total mortality of 75% of the fish was achieved in the control group following challenge. In this group, mortality started one day earlier than the vaccinated groups (Figure 3). There was a significant difference in post challenge survival proportions (PCSPs) between the vaccinated and control groups. Figure 3 shows that there was a dose dependent effect on PCSPs with the HiAg group (8 μg/g of fish body weight), had a significantly higher PCSP (*p* = 0.0435) than the LoAg group. In terms of relative percent survival (RPS), the HiAg group had the highest RPS (79.99%), followed by the LoAg group (RPS = 37.33%) and the NP-Empty (RPS = 3.96%).

#### 3.2.2. Serum Inhibition of *A. hydrophila* Growth on Trypticase Soy Agar (TSA) Agar

**Figure 4:**
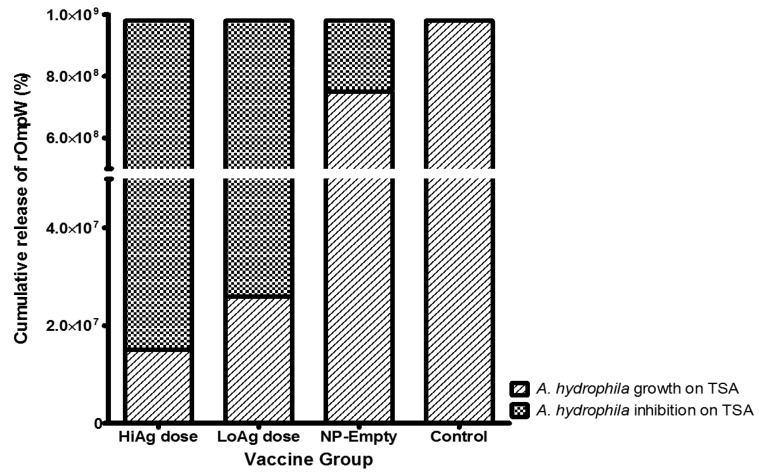
*A. hydrophila* growth and inhibition on trypticase soy agar (TSA) after treatment of sera from fish vaccinated with overnight culture broth of *A. hydrophila*. The *A. hydrophila* culture was used at a concentration of 10^3^ cfu/mL to which 90 μL serially-diluted sera was added in microtubes followed by incubation on TSA for 24 h at 30 °C. *A. hydrophila* growth for each group was determined by counting individual colonies, as described by Lowry *et al.*, while inhibition was calculated by subtracting bacteria colony counts of vaccinated fish from the unvaccinated control group. The serum samples used in this study were pooled from 10 blood samples collected from each group at 50 dpv.

Figure 4 shows results of *A. hydrophila* counts. There was a significant reduction in bacterial counts when bacteria were incubated with serum from vaccinated fish, compared to unvaccinated control fish, with a reduction to less than 2.0 × 10^7^ for HiAg and around 3.0 × 10^7^ for the LoAg group, compared to 1.0 × 10^9^ for the control group. The unvaccinated control fish had higher bacterial growth than fish fed with empty PLGA NPs (Figure 4).

#### 3.2.3. Antibody Responses

**Figure 5:**
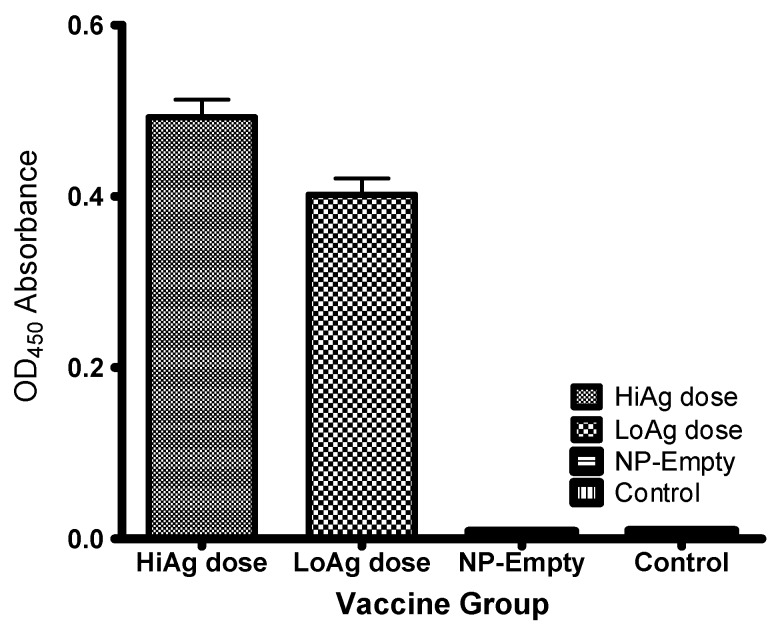
Antibody levels detected against *A. hydrophila* from fish vaccinated with the NP-rOmpW vaccine at HiAg dose, LoAg dose, NP-Empty, and control groups at 51 dpv. Note that antibody levels from fish vaccinated with the HiAg dose were higher than the LoAg dose. There was a significant difference (*p* < 0.0003) in antibody levels detected between the HiAg and LoAg dose groups, while the NP-Empty and control groups did not show presence of antibodies against *A. hydrophila* detected against at 50 dpv.

Figure 5 shows that antibody levels in the control groups (empty-NP and PBS groups) were significantly (*p* < 0.00001) lower than the vaccinated groups (HiAg and LoAg groups). The LoAg group (mean OD_450_ = 0.4160, SD = 0.0017, N = 10) had significantly lower antibody levels (*p* < 0.001) than for the HiAg group (mean OD_450_ = 0.4631, SD = 0.0025, N = 10) indicating that there was an antigen dose-dependency effect on the induction of antibody responses. Both the empty-NP and PBS control group showed no presence of circulating antibodies (Figure 5).

## 4. Discussion

Our findings show that PLGA NP-formulated OmpW used in this study was protective against lethal challenge with *A. hydrophila* in rohu. Further protection against mortality was antigen dose-dependent, *i.e.*, the HiAg group had the highest PCSP, and the LoAg group had a correspondingly low PCSP. This is the first study to document that PLGA NP-formulated OmpWs of *A. hydrophila* confer protective immunity in rohu. The encapsulation efficiency obtained was, however, lower than what has been reported by others [25] and it is, therefore, likely that the quantity of antigen released after oral vaccination *in vivo* was also low, resulting in lower protection than what was obtained from injectable vaccines [27,28,29,30]. Therefore, future studies should seek to increase the content of OmpW encapsulated in PLGA NPs in order to increase the protective ability of the OmpW antigen in vaccinated fish.

Biodegradable PLGA NPs have attracted a lot of interest as an antigen delivery system for oral vaccines because of their ability to enhance antigen uptake and ability to allow the slow release of antigens *in vivo*, an adjuvant depot effect [31,32,33]. The size of the NPs (nanodiameter < 400 nm) generated in this study suggests that these particles are of an adequate size for inducing systemic antibody responses. For systemic responses to occur it is anticipated that uptake and processing of the rOmpW antigens by cells of adaptive immune system for induction of protective immunity is required [31,32,33]. However, the encapsulation efficiency of 53.6% obtained in this study was lower than the mean 60%–70% encapsulation efficiency for PLGA NPs as shown by Kumara *et al.* and Danhier *et al.* [34,35]. Danhier *et al.* [34] observed that high encapsulation efficiencies increase the ability of NPs to deliver antigen *in vivo*; therefore, future studies should seek to increase the encapsulation efficiency of rOmpW in PLGA NPs in order to induce higher protection levels in vaccinated fish.

The OmpW protein belongs to a family of small OMPs that are highly conserved among the Gram-negative bacteria species [36,37] and, being a highly immunogenic protein, able to induce protective immunity in vaccinated fish [27,28,29,30]. Qian *et al*. [30] obtained high protection with a relative percent survival (RPS) of 78 in large yellow croaker (*Pseudosciaena crocea*) when injected intraperitoneally with OmpW against *V. alginolyticus,* while Cai *et al.* [28] showed high protection (RPS = 92) in Crimson snapper (*Lutjanus erythropterus*) following intramuscular injection with OmpW against *V. alginolyticus*. Similarly, Mao *et al.* [30] showed high protection (RPS > 80) in large yellow croaker injected intraperitoneally with OmpW against *V. parahaemolyticus*. Maiti *et al.* [29] obtained high protection (RPS = 80) in carp on intraperitoneal injection with rOmpW against *A. hydrophila*. Results of these studies show that OmpW is protective against different bacteria species when administered by the intramuscular or intraperitoneal route [27,28,29,30]. The results obtained from parenteral delivery of rOmpW of *A. hydrophila* surpass those from oral delivery routes indicating that there is still room for improvement of oral PLGA NP-formulations Determining the ability of PLGA NPs to release the encapsulated antigens in vaccinated fish calls for *in vitro* tests that reflect their ability to release the antigens *in vivo*. In this study, *in vitro* release of rOmpW reached 34% within 48 h after incubation in PBS but was lower than the 50% *in vitro* release obtained by Rauta and Nayak [25] for the same antigen encapsulated in PLGA NPs. The kinetics of rOmpW *in vitro* release obtained in this study follow the common trend that starts with a rapid initial release of antigens within 24 h [25,38], followed by a continuous slow release over a long period. Hence, it is likely that a follow up on the *in vitro* release test carried out in this study after 48 h would have shown a prolonged continuous release of the rOmpW antigen from encapsulated NPs. To evaluate the functional characteristics of elicited antibodies post vaccination we performed a serum inhibition test using serum from vaccinated fish. Our findings show that there was an antigen dose dependency on inhibition of *A. hydrophila* growth on TSA, corresponding to *in vivo* challenge, where serum from the HiAg group had the highest inhibition capacity, lower in the LoAg group. These findings correspond with antibody levels obtained from the vaccinated fish where the HiAg group had the highest antibody levels with the LoAg group having relatively low levels. The NP-Empty and control groups had no circulating antibodies. Thus, antibody levels induced by rOmpW vaccines correspond with vaccine antigen quantity delivered by oral vaccination. These findings show that the serum inhibition test used in this study can be used as an *in vitro* measure of vaccine efficacy, in line with our previous findings in which we showed that antigen dose corresponded with protection in Atlantic salmon vaccinated against IPN [39,40]. Similar observations have been shown for furunculosis vaccines in salmon [41], and also in higher vertebrates in which antigen dose was seen to correlate with the induction of protective immunity. As pointed out in our previous studies [39,40,41], establishing an optimal antigen dose that correlates with protective immunity could serve as a measure of vaccine efficacy for fish vaccines. Hence, future studies should seek to determine the antigen dose and optimal duration of oral vaccination for OmpW that correlate with protective immunity in rohu. In addition, there is a need for detailed investigations to determine the protective mechanism of OmpW oral vaccination, as done for other vaccines [42,43]. There is also a need to elucidate the impact of PLGA NPs in fish vaccinated by the oral route. Nevertheless, this study has shown that OmpW is protective against *A. hydrophila* infection in rohu and that NP-based vaccines could serve as an effective oral immunization strategy for finfish.
